# An image dataset for analyzing tea picking behavior in tea plantations

**DOI:** 10.3389/fpls.2024.1473558

**Published:** 2025-01-15

**Authors:** Ru Han, Ye Zheng, Renjie Tian, Lei Shu, Xiaoyuan Jing, Fan Yang

**Affiliations:** ^1^ School of Computer Science, Guangdong University of Petrochemical Technology, Maoming, China; ^2^ College of Artificial Intelligence, Nanjing Agricultural University, Nanjing, China; ^3^ School of Engineering, University of Lincoln, Lincoln, United Kingdom; ^4^ School of Electrical Engineering and Automation, Jiangsu Normal University, Xuzhou, China

**Keywords:** outdoor scenes, behavior recognition, image data, tea picking, protection of tea plantation

## Abstract

Tea is an important economic product in China, and tea picking is a key agricultural activity. As the practice of tea picking in China gradually shifts towards intelligent and mechanized methods, artificial intelligence recognition technology has become a crucial tool, showing great potential in recognizing large-scale tea picking operations and various picking behaviors. Constructing a comprehensive database is essential for these advancements. The newly developed Tea Garden Harvest Dataset offers several advantages that have a positive impact on tea garden management: 1) Enhanced image diversity: through advanced data augmentation techniques such as rotation, cropping, enhancement, and flipping, our dataset provides a rich variety of images. This diversity improves the model’s ability to accurately recognize tea picking behaviors under different environments and conditions. 2) Precise annotations: every image in our dataset is meticulously annotated with boundary box coordinates, object categories, and sizes. This detailed annotation helps to better understand the target features, enhancing the model’s learning process and overall performance. 3) Multi-Scale training capability: our dataset supports multi-scale training, allowing the model to adapt to targets of different sizes. This capability ensures versatility and accuracy in real-world applications, where objects may appear at varying distances and scales. This tea garden picking dataset not only fills the existing gap in the data related to tea picking in China but also makes a significant contribution to advancing intelligent tea picking practices. By leveraging its unique advantages, this dataset becomes a powerful resource for tea garden management, promoting increased efficiency, accuracy, and productivity in tea production.

## 1 Introduction

As a global leader in tea production, China holds tea not just as an important cash crop, but also as a product leading in market sales worldwide. Tea plantation harvesting stands at the heart in the comprehensive process of tea production, with tea picking as a critical component that is increasingly moving toward intelligence and mechanization (Kisantal et al., 2019). By leveraging smart recognition technologies, we are capable of conducting precise monitoring and management of picking operations in large tea plantation. Specifically:

- To align with the standardized requirements for tea operation procedures and harvesting management, tea plantation can utilize smart recognition technologies to monitor in real time and rectify inappropriate picking behaviors, such as excessive picking or irrational leaf handling, ensuring the stabilization and enhancement of tea quality.
- In tourist-accessible tea plantation, administrators can employ intelligent surveillance systems to ensure that visitors’ picking activities adhere to regulations, maintaining order within the gardens. For those rare ancient tree tea plantation, smart recognition technologies serve as a potent instrument to prohibit picking and protect precious plant specimens. In production-oriented tea plantation, similar monitoring of farmers’ picking behaviors can prevent irregular practices, safeguarding production efficiency and tea quality.
- The tea picking behavior dataset offers researchers in computer vision, machine learning, and deep learning a valuable experimental platform. Researchers can develop and refine algorithms for target detection, behavior recognition, and classification tailored to tea plantation environments, driving the evolution of smart technologies in agriculture.
- Appropriate tea picking in tea plantation can relieve apical dominance in tea plants, promoting continuous bud sprouting on lateral branches. Over-picking, however, can result in too few leaves on the plant, affecting photosynthesis and hindering the formation and accumulation of organic substances, thereby impacting the growth and development of tea plants and the quality of the tea produced.

Tea picking behavior datasets play a crucial role in the development of smart agriculture and automated picking technologies. This dataset provides a wealth of visual information and behavioral patterns by recording and labeling the actual tea picking process, which is critical for training machine learning models to recognize and mimic the movements of human pickers. In this study, the tea picking behavior dataset plays a core role, which lays a foundation for the development of efficient automatic picking algorithms. In addition, such datasets facilitate interdisciplinary collaboration and facilitate communication and integration between computer vision, robotics and agricultural science. In order to identify the behavior of tea plantation picking, the first step is to build dataset. Currently, there is no public dataset of tea plantation picking. A vast array of relevant datasets are widely utilized in domains, e.g., autonomous driving, object detection, facial recognition, semantic segmentation, optical flow, among others. Here are some of the extensively used public datasets:

The nuScenes dataset (Caesar et al., 2019) specifically for pure vision 3D object detection in the autonomous driving sector. The COCO dataset (Rostianingsih et al., 2020; Srivast et al., 2020; Devi and Cn, 2022) contains large-scale common objects for object detection, and datasets tailored for YOLO (Dai et al., 2022; Zhang et al., 2022) optimized for object detection tasks. The VOC dataset (Francies et al., 2021; Varadarajan et al., 2021; Shen et al., 2020) focusing on humans, common animals, vehicles, and indoor furniture for object detection (Cai et al., 2022; Li et al., 2023). The Mapillary Traffic Sign Dataset (Ertler et al., 2020) covering traffic signs across global geographies. The Scale Match (SM) dataset (Yu et al., 2020) designed for small human and small object behavior detection.

Beyond these commonly accessible datasets, custom datasets can be created using frameworks like PyTorch to meet specific requirements (Ignatious et al., 2024). However, creating a custom dataset involves complexities related to formatting, diversity, and interoperability. Thus, when constructing a dataset, considerations must be given to its usability and compatibility to ensure its practicality and promote its wider adoption.

Due to the lack of a comprehensive and publicly available dataset for tea picking behavior, this paper undertook the creation of such a dataset based on an understanding and analysis of the formats and production processes of commonly used object detection and behavior recognition datasets. We have established an image dataset derived from video clips related to tea plantation scenes found on the internet. Spanning videos from 2014 to 2024, the dataset consists of 12,195 sliced picking images featuring five different types of behaviors (list as: 1) pick; 2) pick(machinery); 3) walk; 4) talk; 5) stand) under five distinct environmental conditions: 1) sunny; 2) overcast; 3) cloudy; 4) foggy; 5) rainy. All labels for the dataset are provided in COCO format for public use.

The contributions of this study can be summarized in the following three aspects:

- Creation of the first tea garden picking behavior image dataset: We successfully constructed the Tea Garden Harvest Dataset, which is currently the first image dataset specifically focused on tea garden picking behavior. This dataset not only fills the gap in data resources in this field but also provides valuable data support for intelligent tea garden management and picking behavior analysis.
- High quality and diversity of the dataset: Through carefully designed data augmentation techniques such as rotation, cropping, color enhancement, and flipping, we significantly improved the diversity and quality of the dataset. This ensures that the dataset can support models in accurately recognizing tea picking behaviors under various environmental conditions, thereby enhancing the model’s generalization and practicality.
- Precise annotation and multi-scale training: The images in the dataset have been precisely annotated, including bounding box coordinates, object categories, and sizes. This helps the model better learn and recognize target features. Additionally, the dataset supports multi-scale training, enabling the model to adapt to targets of different sizes, which is crucial for improving the model’s accuracy and adaptability in real-world applications.

These contributions provide important data resources and technical support for the field of smart agriculture, particularly in the area of tea garden picking behavior recognition and analysis, promoting the development of research and applications in this field.

## 2 Value of the data

**Figure 1:**
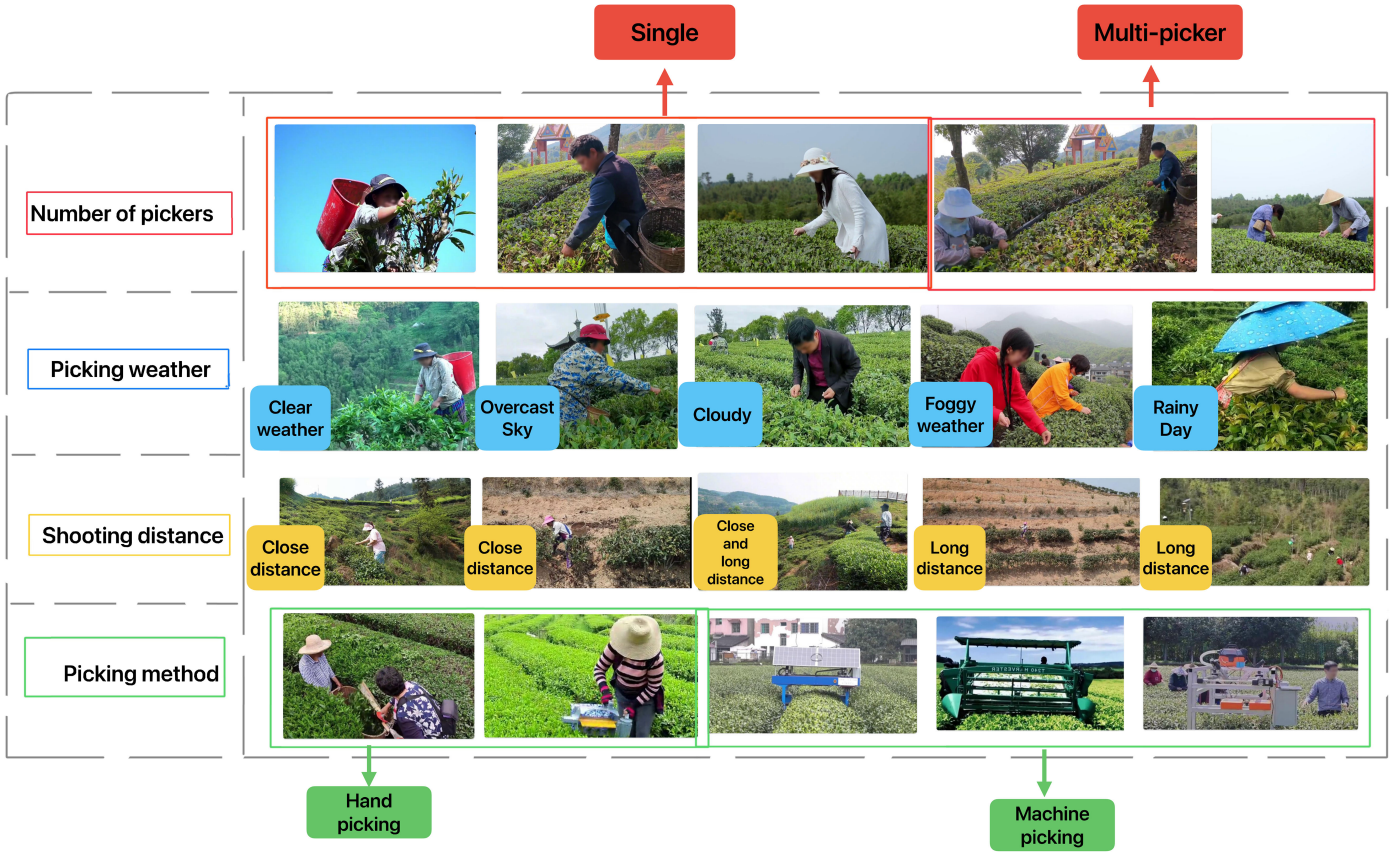
The examples of different picking situations in the dataset include: different number of pickers, different picking weather, different shooting distances, and different picking methods.

1. This paper selected videos about tea picking on the Internet, and crawled most of the tea picking videos on searchable platforms such as Baidu, Good-looking video, Watermelon video, and Bilibili, and sliced the videos. The video scenes include various terrains such as mountains, hills, and plains, and also include various weather conditions: 1) sunny; 2) overcast; 3) cloudy; 4) foggy; 5) rainy. The image video resolutions include 480p, 720p, and 1080p formats, and the video slice sizes include 1920×1280, 854×480, 852×480.
2. The tea picking behavior recognition dataset constructed in this work has a total of seven categories of labels: 1) picking; 2) picking (machine); 3) walking; 4) standing; 5) talking; 6) storage tools; 7) picking tools. Considering that some original images do not contain any of the aforementioned categories during the image slicing process, such images may affect the final judgment. Therefore, while ensuring the continuity of the video as much as possible, we deleted some error-prone image frames and sorted the annotated images to serve as the raw data for the dataset. The **Figure 1** provides a completed list and exemplification of the different conditions in the dataset.

## 3 Materials and methods

### 3.1 Collection and construction of the dataset

**Figure 2:**
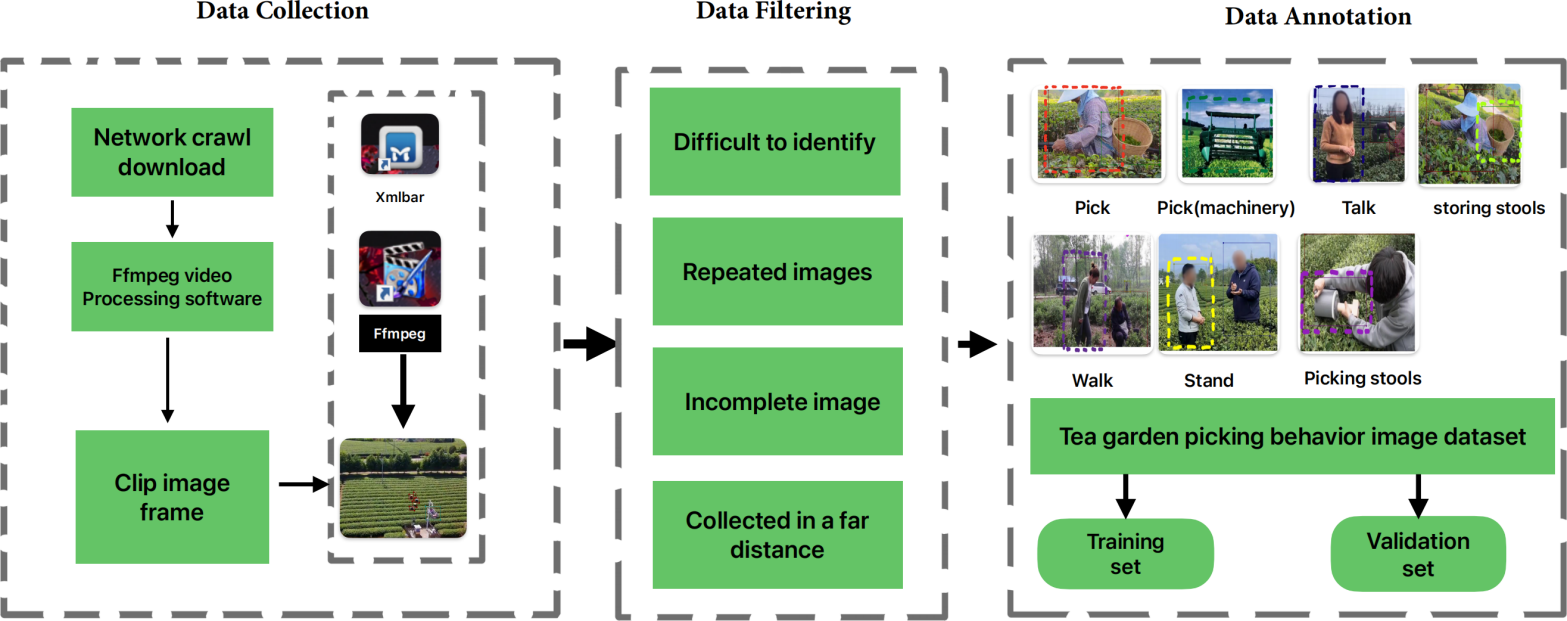
The flowchart of dataset construction.

1. Image acquisition: Our research involves the following content: firstly, we crawl tea picking videos widely available on the internet, including but not limited to major mainstream media and video websites. Secondly, we slice and preprocess the crawled videos to establish a complete dataset that can be used for training and testing. Finally, we use current popular image recognition algorithms to train and achieve recognition requirements. In the annotation process, we noticed that picking, standing, walking, talking, and picking (machine) are common behaviors in current tea picking, while storage tools and picking tools are two commonly used tools, which are also key to tea picking recognition.
2. In the **Figure 2**, the construction process of the tea picking image dataset is as follows. It is divided into three steps: 1) Image data collection. 2) Image data filtering. 3) Image data labeling. Then, the original image dataset is filtered. Since the dataset mainly includes labels for picking, picking (machine), walking, standing, talking, storage tools, and picking tools, these labels require clear and complete images for judgment. However, in the original images, some images are blurry, have poor image quality, unclear or incomplete targets, making it difficult to perform accurate target recognition. Therefore, during the data filtering process, as much as possible, we deleted images that may have abnormal conditions.

The abnormal images that need to be processed mainly include the following situations:

- When the tea bushes block the picking behavior in large areas, it is difficult to recognize the picking behavior in the image.
- When the shooting distance is too far, the targets in the image are too small to distinguish and recognize.
- Due to limited or blocked shooting angles and scenes, the image data obtained after slicing only includes partial features of the labels, resulting in low recognition accuracy.

During the processing of the image dataset, manual or automatic deletion of the above abnormal images is required. At the same time, videos with the same content but different titles on the internet or videos that are contained in other videos are also filtered and deleted.

### 3.2 Data augmentation

Based on the original data, we performed data augmentation operations to expand the dataset and provide more training data. The basic data augmentation methods include rotation, flipping, enhancement, and cropping. The **Table 1** details and lists the data augmentation methods and their corresponding quantities.

**Table 1:** Data augmentation quantity table.

Data enhancement method	Number of pictures
Rotate	3053
Cropping	3113
Enhancement	3015
Flipping	3014

**Figure 3:**
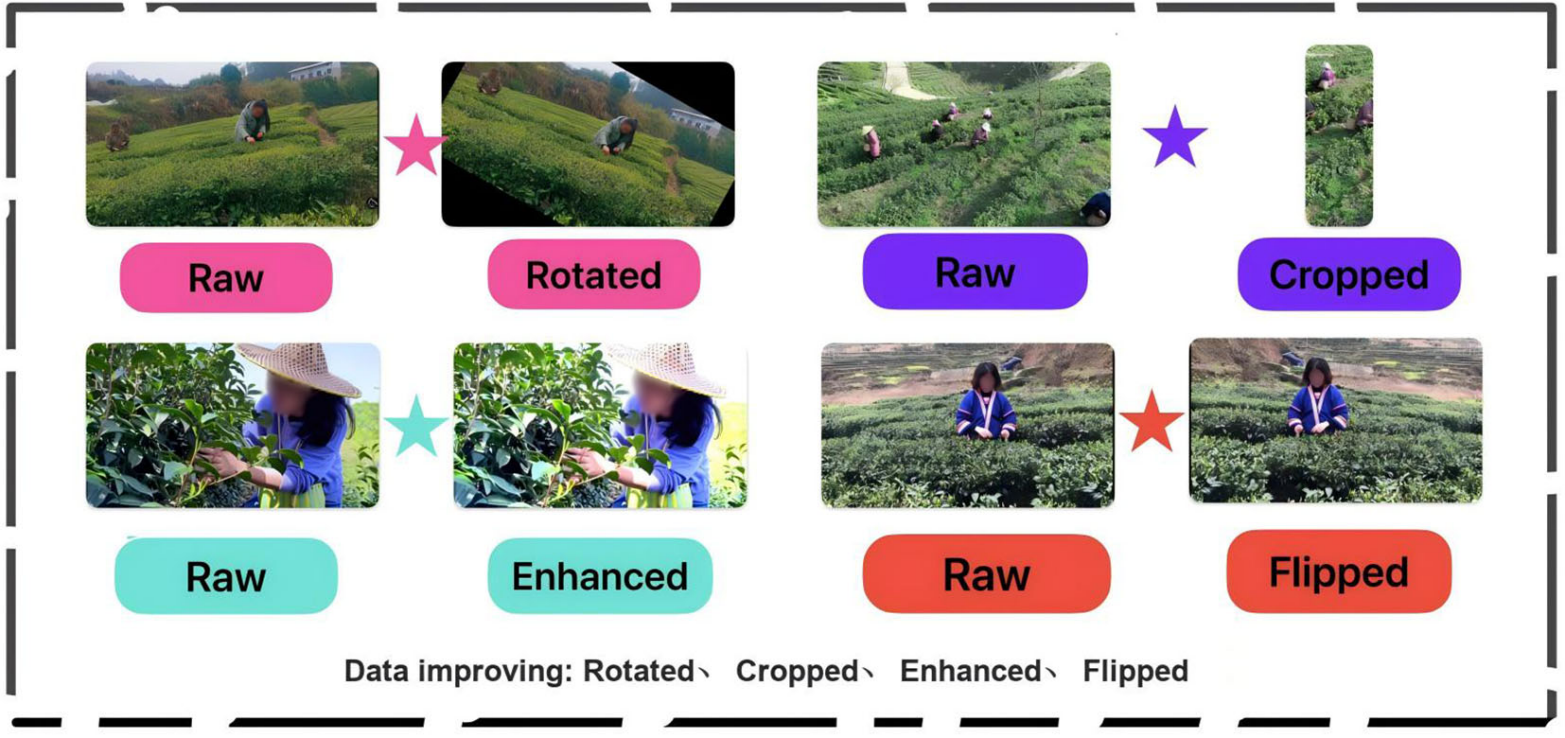
Types of data augmentation and their effect displays.

The **Figure 3** compares the augmented data with the original images and provides examples to demonstrate the augmentation process. During the preprocessing of the dataset, we also noticed that the dataset contains two common challenges in object detection field: small object detection and human behavior recognition, which are prone to errors and result in low model accuracy. Therefore, based on the original dataset, we conducted data augmentation specifically for these two scenarios.

In the section of small object detection, this dataset faces some unique challenges. Due to the widespread and complex camera deployment in the tea plantation environment, there are many objects at a distance, which requires special attention to detect and segment small objects. After observing the label distribution of this dataset, we found the following two characteristics about small objects:

1. Image size: Images containing small objects are relatively small, which leads the model to tend to recognize medium and large-sized objects. Small objects occupy a smaller proportion in the image and are easily overlooked by the human eye and the model.
2. Fewer behavioral features: Most of the small object labels are related to behavior recognition (such as picking tools, storing tools, etc.), which results in less information related to the features of the small objects themselves. Behavior recognition relies more on the overall posture of the object and the surrounding environment rather than subtle details.

### 3.3 Image annotation and dataset production

**Figure 4:**
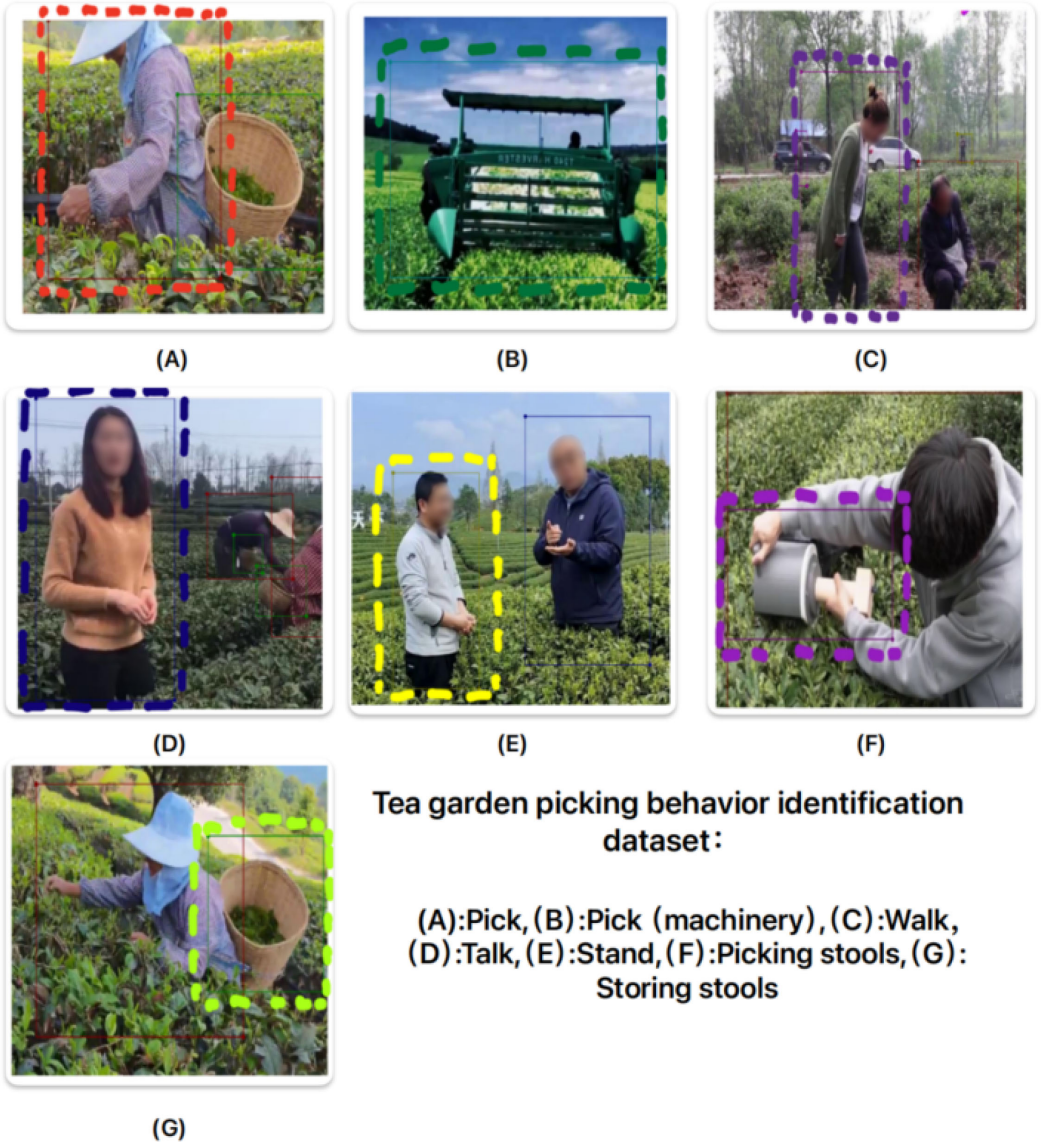
The display of dataset label.

The annotation work is carried out using the labelme software. The staff use labelme to set the labels in the json format and add corresponding annotations based on the video description. The training and validation sets are annotated using labelme, and the json format is converted to COCO format dataset that can be recognized by the model using a script file. The dataset has single-label annotations for picking (human), picking (machine), rest, walking, talking behaviors, and storage and picking tools. The **Figure 4** exemplifies different types of label conditions and annotation methods, In the process of labeling, the small target objects of picking tools and storing tools, as well as the accuracy of object positioning, are taken into account. Therefore, for partially occluded objects, only the visible part is marked to reduce false positives.

To ensure the versatility and compatibility of the dataset, this paper crawled videos on tea picking from multiple scenes on the internet, including different angles (top view, bottom view, side view), different weather conditions (cloudy, sunny, foggy, rainy, overcast), different picking methods (manual, machine), different numbers of people (single person, multiple people), and different distances (long shot, close-up, hand close-up). The scenes involved various regions in China, including the western plain and the eastern tea plantations. The data came from 138 different scenes. The dataset established in this work is divided into a training set, a validation set and a test set in a 7:2:1 ratio.

As shown in **Table 2**, There are 5654 images of single person picking, 6541 images of multiple people picking; 3001 images of sunny weather, 4271 images of cloudy weather, 3852 images of overcast weather, 482 images of foggy weather, and 214 images of rainy weather; 10400 images of close-up picking, 754 images of long shot picking, and 1037 images of both close-up and long shot picking; 11896 images of manual picking, and 299 images of machine picking. It details the different scenarios and conditions in the dataset, as well as the content and quantity of the data.

**Table 2:** Dataset classification and slicing situation table.

Division	Classification	Slice number
Number of pickers	Single	5654
	Multiplayer	6541
Method of picking	Manual	11896
	Machine	299
Picking weather	Sunny	3001
	Overcast	4271
	Cloudy	3852
	Foggy	482
	Rainy	214
Camera distance	Close view	10400
	Distant view	754
	Close+Distant view	1037

### 3.4 Technical validation

**Figure 5:**
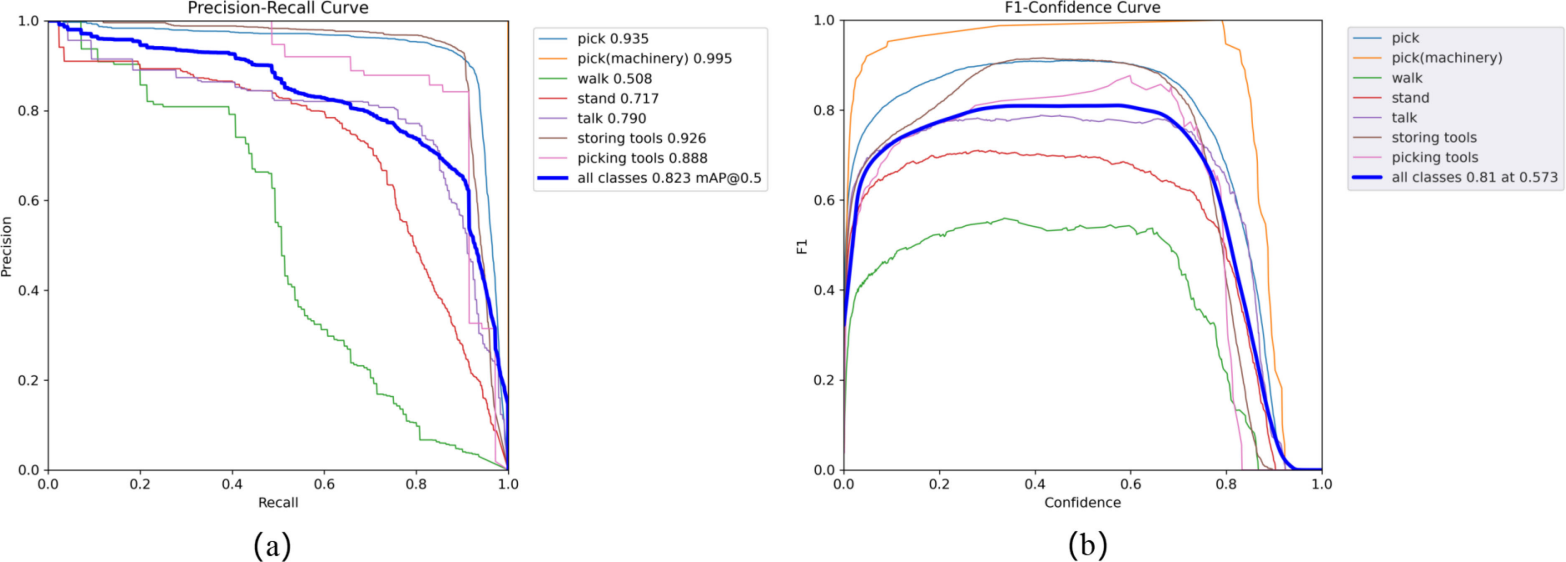
The results of PR curve **(A)** and F1 curve **(B)**.

To ensure the reliability of the tea picking behavior recognition dataset in model training, after the annotation process, we invited experienced professional tea pickers to review and check the labeled images and annotation information. We checked for any missing or incorrect annotations and obtained the final dataset. As shown in **Figure 5**, PR curve and F1 curve obtained by training with Yolov5s as the pre-trained weights. We can obtain the recognition accuracy of various annotated behaviors in the tea plantation picking scenario.

**Figure 6:**
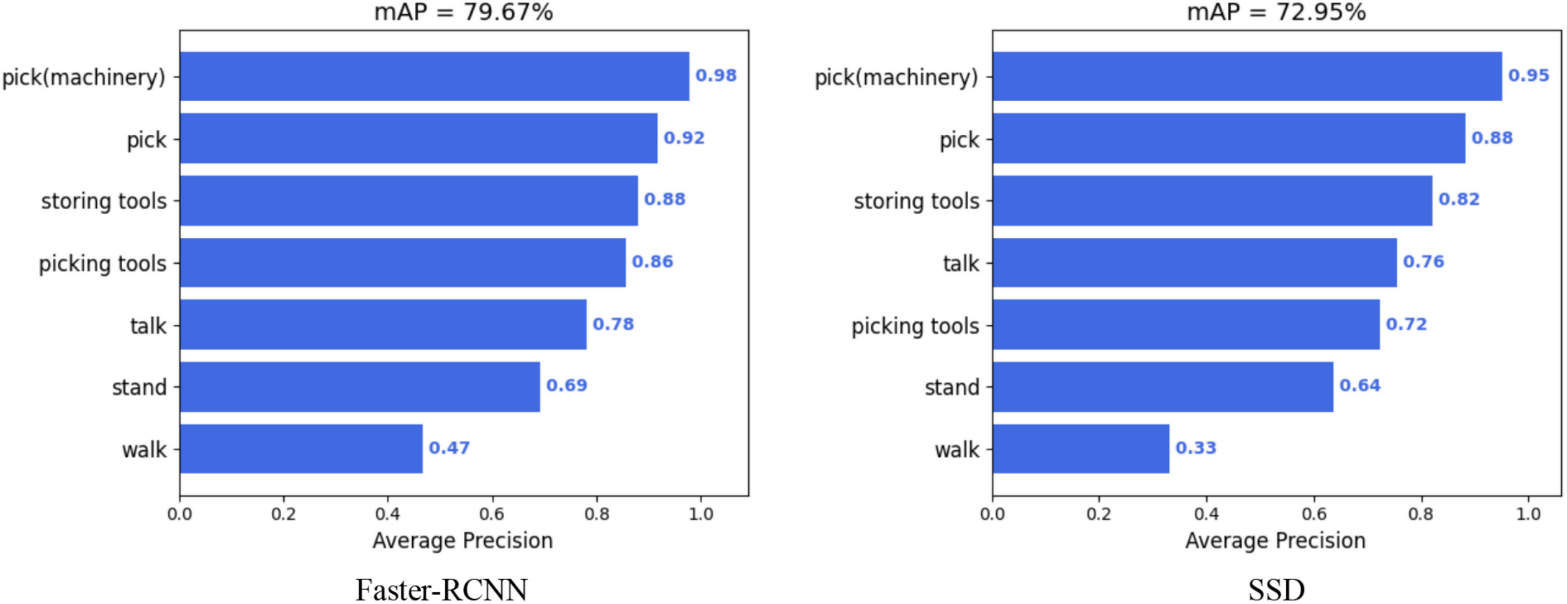
The results of mAP50 in Faster-RCNN and SSD.

In the experimental validation phase using the tea garden picking dataset, to comprehensively evaluate the performance of this dataset, two commonly used object detection networks, Faster R-CNN and SSD, were also selected. These networks were tested on multiple tasks, and the model’s mean average precision (mAP) was calculated to assess their performance, as shown in **Figure 6**.

Experimental results showed Faster R-CNN achieved the highest mAP (79.67%), demonstrating strong recognition of tea garden machinery. SSD had a slightly lower mAP (72.95%) but still recognized well. However, performance varied by task; both networks excelled on Storing tools and Talk but struggled on Stand and Walk, with SSD’s mAP on Walk being only 0.33%.

The performance of the tea garden picking dataset varies across tasks, potentially due to dataset characteristics and network structures/training strategies. Future research should explore improved network structures and training methods to enhance recognition accuracy and generalization. Moreover, incorporating more datasets and tasks can enrich the field of tea garden picking research.

As shown in **Table 3**, We evaluated the performance of three popular object detection algorithms (Faster R-CNN, SSD, and YOLOv5s) on the tea garden picking dataset. By comparing their precision (P), recall (R), and mean average precision at 50% IoU (mAP50), we can come to the following conclusions:

- YOLOv5s performed exceptionally well across all three metrics, achieving the highest precision (84.5%), recall (78.8%), and mAP50 (82.3%). This indicates that YOLOv5s not only accurately identifies targets in tea garden picking scenarios but also covers a larger number of true targets while maintaining high overall detection performance.
- SSD exhibited the best precision (81.90%) but fell short in recall (66.80%), resulting in an mAP50 of 72.95%. This suggests that SSD might miss some targets in certain situations, despite its high accuracy in identifying targets.
- Faster R-CNN excelled in recall (81.72%) but had a relatively lower precision (67.56%), leading to more false positives. Its mAP50 of 79.67% indicates challenges in balancing precision and recall.

**Table 3:** A comparative analysis of the experimental outcomes from various target detection algorithms.

Model	P	R	mAP50
Faster R CNN	67.56	81.72	79.67
SSD	81.90	66.80	72.95
YOLOv5s	84.5	78.8	82.3

In summary, YOLOv5s balanced precision and recall best on the tea garden picking dataset, suitable for high-performance applications. SSD excelled in precision but required recall optimization. Faster R-CNN had high recall but lower precision in some cases. These findings guide algorithm selection for tea garden picking tasks.

Synthesize above, this article establishes a dataset from online video slices of tea plantation scenes. The dataset spans 12,195 images of tea picking from online videos related to tea plantation picking from 2014 to 2024, including five different behaviors and two different tools. The labels of the dataset are publicly provided in COCO format.

## Data availability statement

Publicly available datasets were contributed in this study. This data can be found at: https://dx.doi.org/10.21227/dnkh-8e73.
