# Corrigendum: An image dataset for analyzing tea picking behavior in tea plantations

**DOI:** 10.3389/fpls.2025.1582905

**Published:** 2025-04-23

**Authors:** Ru Han, Ye Zheng, Renjie Tian, Lei Shu, Xiaoyuan Jing, Fan Yang

**Affiliations:** ^1^ School of Computer Science, Guangdong University of Petrochemical Technology, Maoming, China; ^2^ College of Artificial Intelligence, Nanjing Agricultural University, Nanjing, China; ^3^ School of Engineering, University of Lincoln, Lincoln, United Kingdom; ^4^ School of Electrical Engineering and Automation, Jiangsu Normal University, Xuzhou, China

**Keywords:** outdoor scenes, behavior recognition, image data, tea picking, protection of tea plantation

In the published article, there were typos in Figures 1–3 as published. There was a typo in the original **Figure 1**, “Close Disatnce” and “Long Disatnce” have been corrected to “Close Distance” and “Long Distance”. The corrected Figure 1 is shown below. There was a typo in the original **Figure 2**, “Data Laeling” has been corrected to “Data Annotation”. The corrected **Figure 2** is shown below. There was a typo in the original **Figure 3**, “Data improving: Roated, Cropped, Enhanced, Flipped” has been corrected to “Data improving: Rotated, Cropped, Enhanced, Flipped”. The corrected **Figure 3** is shown below.

**Figure 1:**
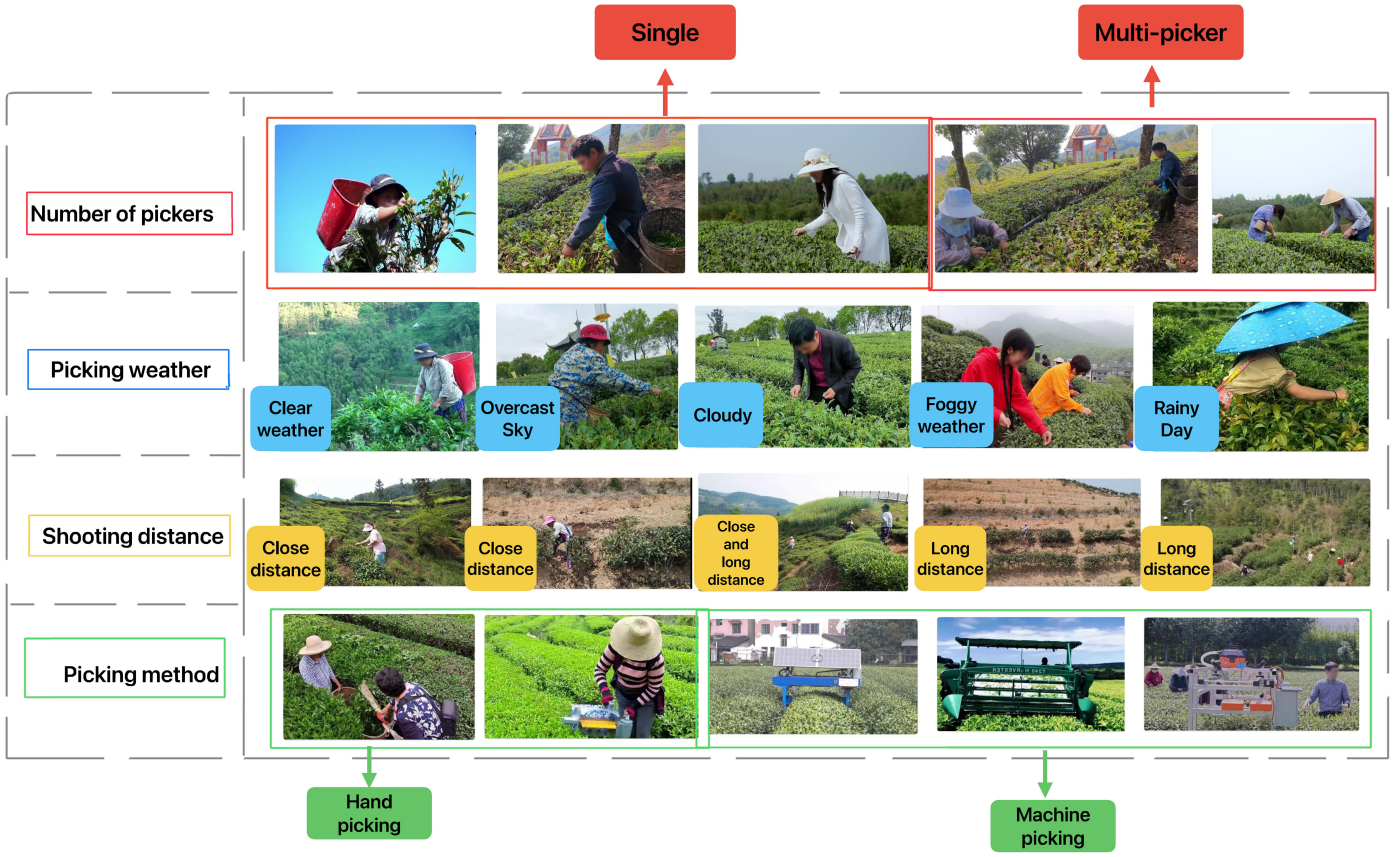
The examples of different picking situations in the dataset include: different number of pickers, different picking weather, different shooting distances, and different picking methods.

**Figure 2:**
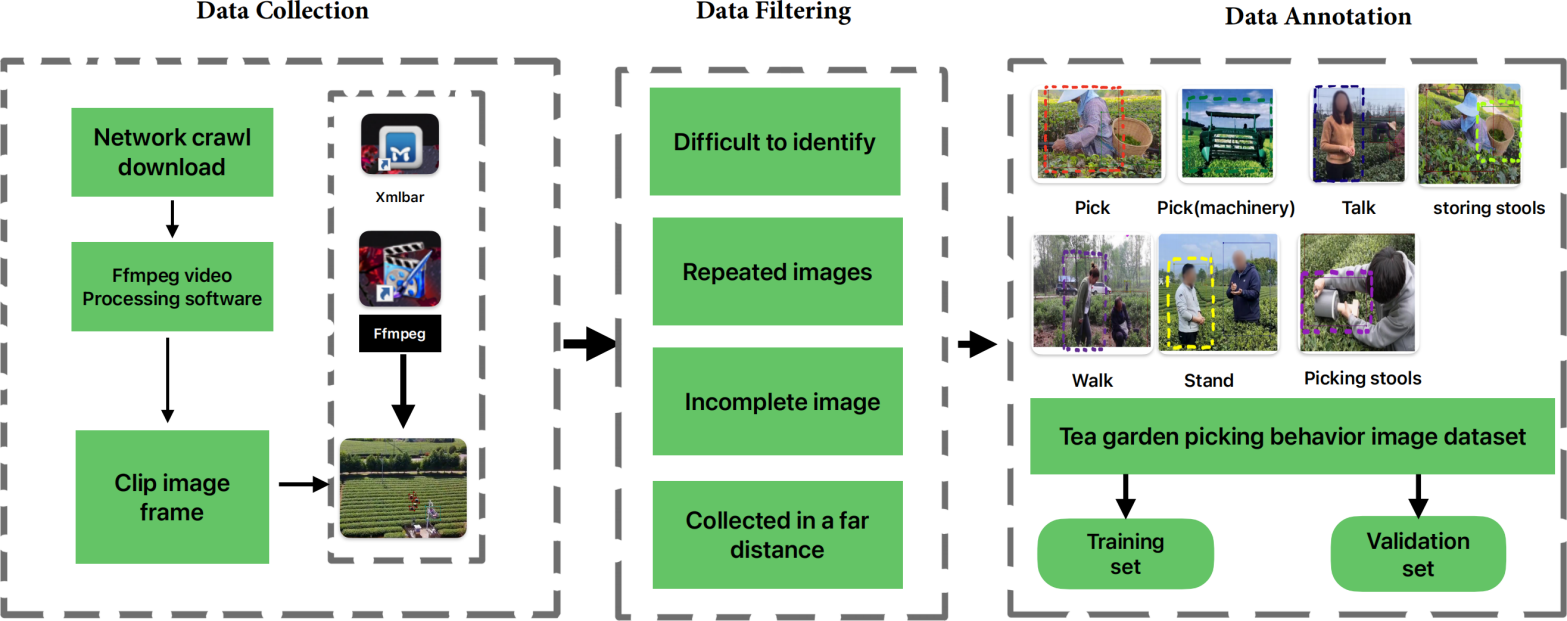
The flowchart of dataset construction.

**Figure 3:**
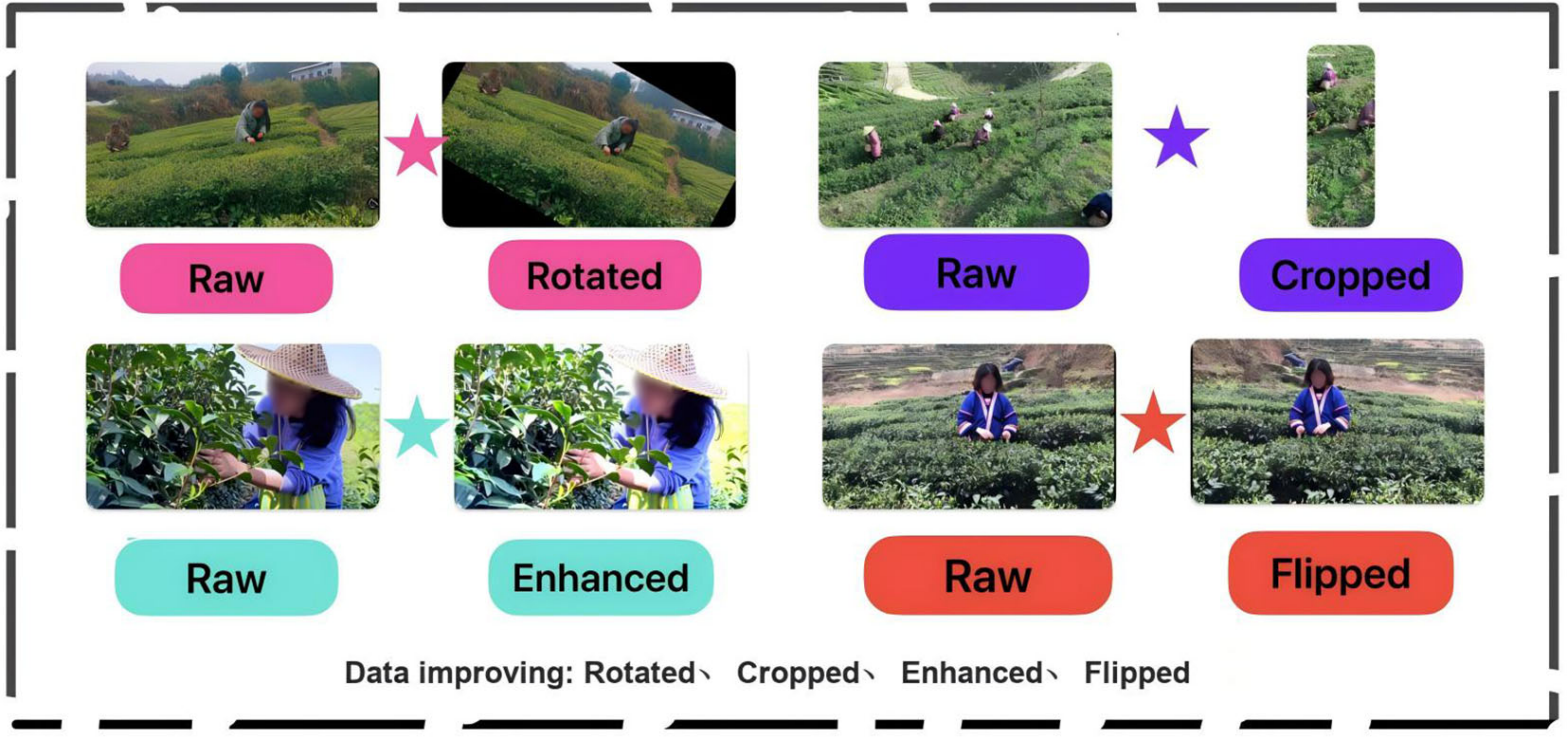
Types of data augmentation and their effect displays.

The authors apologize for these errors and state that this does not change the scientific conclusions of the article in any way. The original article has been updated.

